# Dynamic Increase in Corticomuscular Coherence during Bilateral, Cyclical Ankle Movements

**DOI:** 10.3389/fnhum.2017.00155

**Published:** 2017-04-04

**Authors:** Takashi Yoshida, Kei Masani, Karl Zabjek, Robert Chen, Milos R. Popovic

**Affiliations:** ^1^Rehabilitation Engineering Laboratory, Toronto Rehabilitation Institute, University Health NetworkToronto, ON, Canada; ^2^Institute of Biomaterials and Biomedical Engineering, University of TorontoToronto, ON, Canada; ^3^Department of Physical Therapy, University of TorontoToronto, ON, Canada; ^4^Division of Neurology, Department of Medicine, University of TorontoToronto, ON, Canada; ^5^Krembil Research Institute, University Health NetworkToronto, ON, Canada

**Keywords:** corticomuscular coherence, motor control, locomotion, electroencephalography, electromyography

## Abstract

In humans, the midline primary motor cortex is active during walking. However, the exact role of such cortical participation is unknown. To delineate the role of the primary motor cortex in walking, we examined whether the primary motor cortex would activate leg muscles during movements that retained specific requirements of walking (i.e., locomotive actions). We recorded electroencephalographic and electromyographic signals from 15 healthy, young men while they sat and performed bilateral, cyclical ankle movements. During dorsiflexion, near-20-Hz coherence increased cyclically between the midline primary motor cortex and the co-contracting antagonistic pair (i.e., tibialis anterior and medial gastrocnemius muscles) in both legs. Thus, we have shown that dynamic increase in corticomuscular coherence, which has been observed during walking, also occurs during simple bilateral cyclical movements of the feet. A possible mechanism for such coherence is corticomuscular communication, in which the primary motor cortex participates in the control of movement. Furthermore, because our experimental task isolated certain locomotive actions, the observed coherence suggests that the human primary motor cortex may participate in these actions (i.e., maintaining a specified movement frequency, bilaterally coordinating the feet, and stabilizing the posture of the feet). Additional studies are needed to identify the exact cortical and subcortical interactions that cause corticomuscular coherence and to further delineate the functional role of the primary motor cortex during bilateral cyclical movements such as walking.

## Introduction

Traditionally, it is thought that basic patterns of locomotion are controlled primarily by subcortical and spinal networks (Takakusaki et al., 2004; Pearson and Gordon, 2013). However, recent functional neuroimaging studies in humans have shown that the midline (i.e., the most medial) primary sensorimotor cortex is significantly active during steady-state walking (Fukuyama et al., 1997; Hanakawa et al., 1999; Miyai et al., 2001; Wagner et al., 2012, 2014; Seeber et al., 2014, 2015; Storzer et al., 2016). Specifically, within the gait cycle, the midline primary sensorimotor cortex cyclically increases its activity approximately between mid-beta and low-gamma frequencies (Wagner et al., 2012, 2014; Seeber et al., 2014, 2015; Storzer et al., 2016). Furthermore, Petersen et al. (2012) have reported that, during treadmill walking, the activities of the midline primary motor cortex and the foot dorsiflexor become cyclically coherent, with similar timing and frequency range as the aforementioned increase in the midline sensorimotor activity. Such coherence (i.e., corticomuscular coherence) may indicate corticospinal activation of the muscle (Ushiyama et al., 2011b). Thus, the above findings suggest that the human primary motor cortex participates in steady-state locomotion, perhaps cyclically via the corticospinal tract.

To our knowledge, the study by Petersen et al. (2012) is the only one that investigated corticomuscular coherence during bipedal locomotion. However, bipedal locomotion is a complex task that requires maintenance of a specific movement frequency, balance with full weight bearing, visuomotor integration, and coordination of multi-joint movements. Therefore, during bipedal locomotion, it is uncertain which aspect of locomotor control is represented by corticomuscular coherence.

The purpose of this study was to investigate whether bilateral, cyclical ankle movements involved corticospinal activation of muscles, assuming that such activation could be quantified by corticomuscular coherence. Simplifying the movement eliminated many requirements of bipedal locomotion and increased the probability that the observed coherence was relevant to specific locomotive actions (i.e., maintenance of rhythm and bilateral coordination of the feet). The simplicity of the movement also reduced the risk of motion artifacts. By examining corticomuscular coherence during simple movements, we aimed to better elucidate how the primary motor cortex participates in the control of bipedal locomotion. To our knowledge, there is no study that describes dynamic changes in corticomuscular coherence during simple cyclical leg movements, as previous studies have overwhelmingly focused on sustained contractions of various upper- and lower-limb muscles (Conway et al., 1995; Salenius et al., 1997; Brown et al., 1998; Kilner et al., 1999, 2000; Gross et al., 2000; Kristeva-Feige et al., 2002; Riddle and Baker, 2006; Omlor et al., 2007, 2011; Masakado and Nielsen, 2008; Chakarov et al., 2009; Johnson et al., 2011; Ushiyama et al., 2011a,b, 2012; Gwin and Ferris, 2012; McClelland et al., 2012; Ushiyama, 2013; Trenado et al., 2014). A few studies have examined dynamic movements, but the movements were discrete and ballistic (Muthuraman et al., 2012) or phasic but much slower than walking (Brown et al., 1998).

In previous studies that reported corticomuscular coherence during sustained muscle contractions, the maximum increase in coherence was usually observed around 13–30 Hz (i.e., near the β band) (Conway et al., 1995; Salenius et al., 1997; Brown et al., 1998; Kilner et al., 1999, 2000; Gross et al., 2000; Kristeva-Feige et al., 2002; Riddle and Baker, 2006; Omlor et al., 2007, 2011; Masakado and Nielsen, 2008; Chakarov et al., 2009; Johnson et al., 2011; Ushiyama et al., 2011a,b, 2012; McClelland et al., 2012; Ushiyama, 2013; Trenado et al., 2014). Also, such coherence showed somatotopy: the maximum coherence was observed between the contracting muscle and the corresponding area of the primary motor cortex (Conway et al., 1995; Salenius et al., 1997; Brown et al., 1998; Kilner et al., 1999; Gross et al., 2000). In walking, coherence increased dynamically within the movement cycle, coinciding with increased muscle activity (Petersen et al., 2012). Therefore, we hypothesized that, during cyclical ankle movements, corticomuscular coherence would (i) occur near the β band; (ii) show somatotopy; and (iii) increase dynamically within the movement cycle, coinciding with increased muscle activity. We further hypothesized that, between the tibialis anterior and medial gastrocnemius muscles, corticomuscular coherence would be observed only for the tibialis anterior muscle, as it was the agonist of the movement and had a stronger corticospinal connection (Brouwer and Ashby, 1992). Finally, we hypothesized that rhythmic aural pacing would increase the participant's attention to the movement, resulting in corticomuscular coherence with greater magnitude. This hypothesis was based on the findings of previous studies that increased attention or effort increased corticomuscular coherence (Kilner et al., 2000; Riddle and Baker, 2006; Kristeva et al., 2007; Masakado and Nielsen, 2008; Chakarov et al., 2009; Omlor et al., 2011; Ushiyama et al., 2011a; McClelland et al., 2012; Trenado et al., 2014). Therefore, the cyclical movements were performed under two conditions: (i) self-paced and (ii) externally paced by the sound of a metronome.

## Materials and methods

### Participants

Fifteen men were recruited by convenience sampling. They were 26.7 ± 7.4 years old, 177 ± 7 cm tall, and 74.9 ± 11.0 kg in weight. All participants were able to walk unassisted and reported no history of neurological disorders. The participants were not screened for the presence of corticomuscular coherence before the experiment. Before participating in this study, all participants provided their written informed consent. The experimental protocol had been approved by the University Health Network Research Ethics Board, Toronto, Canada, and they were performed according to the relevant guidelines.

### Experimental task

**Figure 1:**
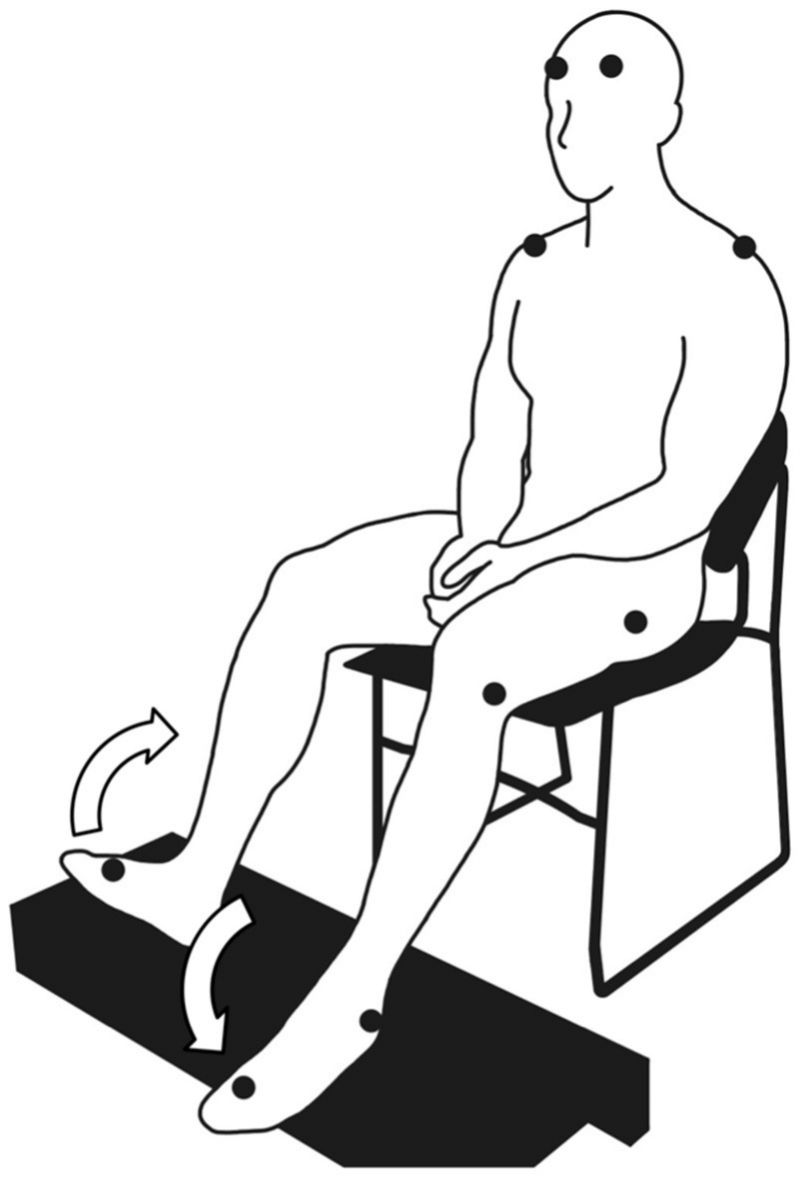
**Posture assumed by the participants to perform cyclical ankle movements**. The symbol, •, indicates the placement of the markers for the motion capture system (where visible). The arrows indicate the anti-phasic ankle movements.

Each participant sat in a chair with a backrest and placed their feet on a footrest (Figure 1). In this position, the participants performed six runs of cyclical ankle movements. Each run lasted approximately 1 min and preceded a rest. The ankle movements were performed under two conditions: (i) self-paced and (ii) externally paced by the sound of a metronome. Each run alternated between self-paced and externally-paced movements, with the first run always being externally paced. The alternation between the two types of pacing was similar to the design of previous studies, which examined the ability to perform self-paced cyclical movements (Ivry and Keele, 1989; Harrington et al., 1998). We did not randomize the order of self- and externally-paced runs because the resultant inter-run and inter-individual variabilities of movement cycle duration could have been too large to ensemble average the runs for each participant or compare the ensemble averages between participants. When the movements were externally paced, the participants were instructed to maximally dorsiflex one foot and maximally plantarflex the other foot at each beat of the metronome. Thus, the instances of maximum and minimum dorsiflexion alternated between the two feet. The metronome was set to 108 beats per minute, which was comparable to the cadence of normal overground locomotion (Murray et al., 1978). For self-paced movements, the participants were instructed to maintain the same rhythm as the externally-paced movements. Because the participants' feet were elevated (Figure 1), the soles of their feet largely did not come in contact with any surface during the movements.

To perform the ankle movements, the participants were instructed to flex or extend their entire foot at the ankle without flexing or extending their toes. The participants were also instructed to maintain a consistent rhythm and to focus their gaze on a bullseye, which was placed in their line of sight as they sat upright and gazed forward. To minimize the source of artifacts in EEG signals, the participants were instructed to relax their upper body and to refrain from moving their head, talking, swallowing, coughing, clenching their jaw, and blinking excessively. While the participants performed the cyclical ankle movements, their EEG signals, EMG signals, and kinematic data were recorded.

### Data collection

All signals were recorded in 1-min epochs. Each epoch began after the experimenter visually confirmed that the participant had started the movement in rhythm. The participant was told to stop the movement after the recording had stopped.

#### Kinematic data

We used an optical motion capture system to track the participants' movements. The system comprised a data acquisition device (MX Giganet, Vicon Motion Systems Ltd., Oxford, United Kingdom), nine optical cameras (Bonita, Vicon Motion Systems Ltd., Oxford, United Kingdom), and data acquisition software (Nexus 1.8.5, Vicon Motion Systems Ltd., Oxford, United Kingdom). Using double-sided adhesive tape, we placed 14-mm retroreflective markers over various bony landmarks, which were identified by manual palpation (Figure 1). The participants wore socks and a tight-fitting outfit, which reduced the movements of the markers with respect to their skin and minimally obscured the markers. The markers over the spinous process of the seventh cervical vertebra and acromio-clavicular joints were placed on the skin. The markers over the greater trochanters, lateral epicondyles of the femur, lateral malleoli, and second metatarsal heads were placed on the outfit. To track head movements, markers were placed over the EEG electrode locations, AF_7_ and AF_8_ (American Electroencephalographic Society, 1991). Except for the one over the cervical vertebra, markers were placed bilaterally. The instantaneous positions of the markers were sampled at 100 Hz.

#### EMG signals

We used a wireless EMG system to record the EMG signals (Trigno™ Wireless EMG System, Delsys Inc., Natick, MA). Each EMG sensor used 99.9%-silver parallel-bar electrodes, which were 1 mm in diameter, 5 mm in length, and spaced at 10 mm. Before placing the EMG sensors, we removed hair from the target location and exfoliated the skin. Then, we used double-sided adhesive tape to place the EMG sensors bilaterally over the bellies of the tibialis anterior and medial gastrocnemius muscles. EMG signals were sampled at 2 kHz, with a bandwidth of 20–450 Hz and the common mode rejection ratio of over 80 dB. EMG signals were sampled by the same software as the motion capture system.

#### EEG signals

We used an active electrode system to record the EEG signals (g.GAMMAsys, g.tec medical engineering GmbH, Schiedlberg, Austria) with compatible signal amplifiers (g.USBamp, g.tec medical engineering GmbH, Schiedlberg, Austria) and recording software (g.Recorder, g.tec medical engineering GmbH, Schiedlberg, Austria). We used a cap (g.GAMMAcap^2^, g.tec medical engineering GmbH, Schiedlberg, Austria) to record EEG signals from 20 locations: AF_z_, F_*z*_, F_1_, F_2_, F_3_, F_4_, FC_z_, FC_1_, FC_2_, FC_3_, FC_4_, C_z_, C_1_, C_2_, C_3_, C_4_, CP_z_, CP_1_, CP_2_, and P_z_, according to the 10-10 system (American Electroencephalographic Society, 1991). This configuration of electrodes covered the midline sensorimotor cortices and their vicinity. We used conductive gel to establish skin-to-electrode contact. The signals were recorded using a monopolar montage with the reference electrode on the left ear lobe and the ground electrode over the right zygomatic process of the temporal bone. EEG signals were sampled at 1.2 kHz without filtering. We used an analog switch to timestamp the EEG signal, and the same switch triggered the sampling by the motion capture system, which also collected EMG signals.

### Data analysis

All calculations were performed in a commercial numerical computing environment (MATLAB R2014b, The MathWorks, Inc., Natick, MA).

#### Motor performance

Performance of the ankle movements was evaluated using the intra-individual mean and standard deviation of the movement cycle duration and range of motion at the ankle. For each participant, the mean and standard deviation were calculated across all movement cycles, with each cycle defined by two consecutive local maxima in the vertical elevation of the motion-capture marker over the second metatarsal head of the right foot. In other words, dorsiflexion on the right was maximal at the beginning and end of each cycle. The ankle angle was calculated between the shank and the foot. The shank was defined as a line between the markers over the lateral epicondyle of the femur and the lateral malleolus, and the foot was defined as a line between the markers over the lateral malleolus and the second metatarsal head. To measure head movements within each movement cycle, we calculated the linear movements of the markers at the EEG electrode locations, AF_7_ and AF_8_.

#### EMG and EEG signals

For both EMG and EEG signals, each 1-min recording was processed separately. The EMG signals were centered and then full-wave rectified. The EEG signals were first filtered by (i) a second-order infinite impulse response notch filter with a center frequency of 60 Hz and bandwidth of 1 Hz and (ii) a fourth-order Butterworth infinite impulse response filter with a passband between 0.5 and 100 Hz. For both processes, zero-phase digital filtering was used. After filtering, the EEG signals were decomposed by independent component analysis using the algorithm by Hyvärinen (1999) and Hyvärinen and Oja (2000). This decomposition isolated artifacts to one or a few independent components. The filtered EEG signals and their independent components were visually inspected for artifacts. During the visual inspection, artifacts were identified based on two characteristics: (i) waveform and (ii) biological plausibility (Libenson, 2010). Some artifacts were identified based on their waveforms. Such artifacts included electrooculographic artifacts, EMG artifacts, and ECG artifacts. Other artifacts were identified by their biological implausibility. For any deflection in an EEG signal, its biological plausibility can be determined based on topography and polarity (Libenson, 2010). Topography describes how the amplitude of a deflection changes over the scalp: if the deflection is caused by a biological event, its amplitude should be maximum at a certain point on the scalp and decay with various gradients away from that point. Also, the polarity of such a deflection should not change over the scalp. Based on these principles, any biologically implausible deflection was considered an artifact. The contributions of independent components that contained artifacts were subtracted from the filtered EEG signals to produce noise-reduced EEG signals. This subtraction was restricted to the observed duration of the artifactual waveform to minimize the loss of information.

#### EEG-EMG coherence

EEG-EMG coherence was calculated for both the tibialis anterior and medial gastrocnemius muscles using wavelet analysis. Wavelet analysis enabled us to study dynamic changes in EEG-EMG coherence over specific frequency bands (i.e., as three-dimensional data). EEG-EMG coherence was calculated separately for each 1-min recording. First, the noise-reduced EEG signals and rectified EMG signals were down-sampled at 400 Hz, and their wavelet coherence was calculated using the following equation (wcoher, Wavelet Toolbox):

$$\frac{{\left|S\left(C_{x}^{*}(a,b)C_{y}(a,b)\right)\right|}^{2}}{S\left({\left|C_{x}(a,b)\right|}^{2}\right)S\left({\left|C_{y}(a,b)\right|}^{2}\right)},$$

where *x* and *y* are two one-dimensional time series, *S* is the smoothing operator in time, the asterisk indicates a complex conjugate, and *C*_*x*_(*a,b*) and *C*_*y*_(*a,b*) are respectively the continuous wavelet transforms of *x* and *y*. Smoothing was applied using a moving average filter with the window length of 200 data points. The continuous wavelet transform calculated by the following equation:

$$C_{x}(a,b)=\int_{-\infty }^{\infty }x(t)\frac{1}{\sqrt{a}}\psi^{*}\left(\frac{t-b}{a}\right)dt,$$

where *x*(*t*) is the time series, whose transform is calculated; ψ is the analyzing wavelet; and *a* is the scale of the analyzing wavelet at position, *b*, in time. The scale, *a*, is related to frequency, *f*, by the following equation:

$$f=\frac{F_{c}}{a\Delta t_{,}}$$

where *F*_*c*_ is the center frequency of the analyzing wavelet and Δ*t* is the sampling interval. For the analyzing wavelet, the complex Morlet wavelet was used:

$$\psi (t)=F_{b}\pi^{-0.5}e^{j2\pi F_{c}t}e^{-\frac{t^{2}}{F_{b}}},$$

where *j* is the imaginary unit, *F*_*b*_ is a bandwidth parameter, and *F*_*c*_ is the center frequency of the wavelet in Hz. The bandwidth parameter and center frequency were set to 10 and 1, respectively. For each participant, an ensemble average of EEG-EMG coherence was calculated by segmenting the coherence into individual movement cycles. The ensemble average was calculated for all EEG electrode locations.

#### Magnitude and frequency of EEG-EMG coherence

**Figure 2:**
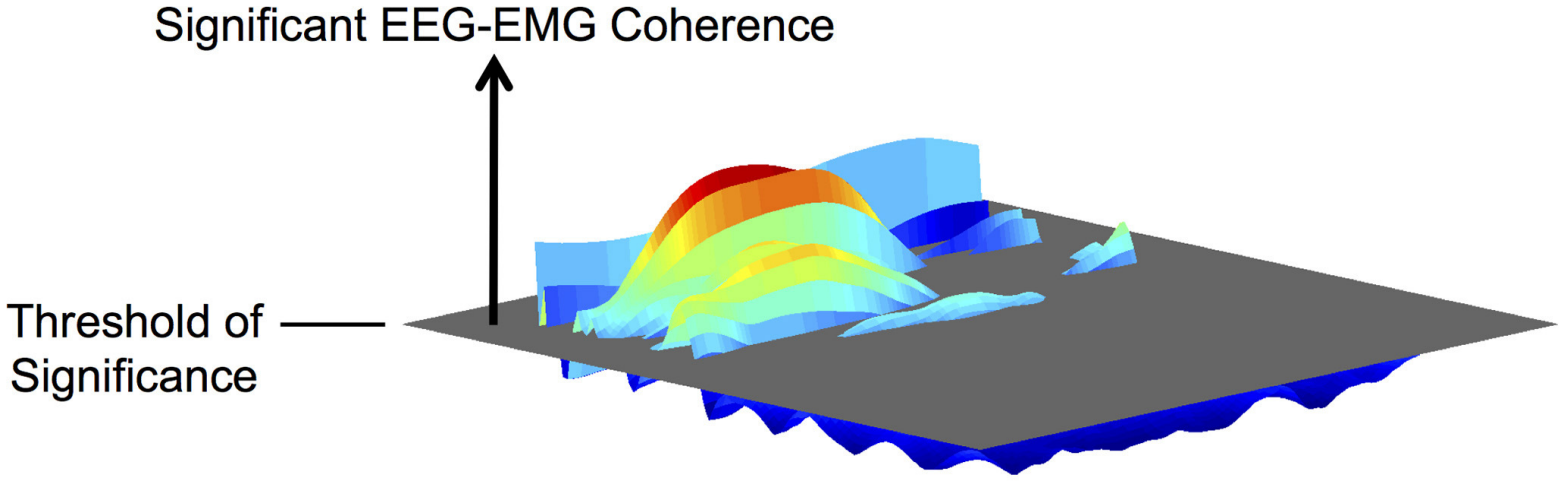
**Illustration of the volume of EEG-EMG coherence above the threshold of significance on the frequency-time plane**.

We quantified the magnitude and frequency of coherence as the volume of significant EEG-EMG coherence and its center frequency, respectively, on the frequency-time plane (Figure 2). Previous studies have typically quantified coherence without temporal resolution (i.e., as two-dimensional data) (Brown et al., 1998; Riddle and Baker, 2006; Kristeva et al., 2007; Masakado and Nielsen, 2008; Johnson et al., 2011). This approach is appropriate for quantitative analysis of coherence during sustained muscle contractions because the cortical participation can be assumed as relatively steady. However, for cyclical movements, it is more intuitive to consider the temporal modulation of coherence within each movement cycle. Thus, we quantified EEG-EMG coherence by its volume above the threshold of significance on the frequency-time plane. A similar approach has been used by Kilner et al. (2000).

Before evaluating significance, each ensemble average of EEG-EMG coherence was binned across frequency and time: binning across frequency resulted in one pixel per Hz between 1 and 100 Hz; binning across time resulted in effective sample frequency of 100 Hz. The threshold of significance, *SL*, was calculated using the following equation (Ushiyama et al., 2012):

$$SL=1-{\left[\frac{1}{N}\left(1-\frac{\alpha }{100}\right)\right]}^{\frac{1}{L\;-\;1}},$$

where α is the confidence level in percent, *L* is the number of disjoint segments that are used to estimate the cross spectra of the EEG and EMG signals, and *N* is the number of observations (i.e., the number of pixels in the binned coherence). The above equation accounts for the multiple observations across frequency and time by using the Bonferroni correction. Our confidence level was 95%. For *L*, we used the number of movement cycles that each participant completed. Using the above threshold, we calculated the volume of significant coherence at each EEG electrode location of each participant. The volume was measured in Hz multiplied by the percentage of movement cycle (Hz • %_*Movement Cycle*_) and calculated above 6 Hz to exclude the low-frequency coherence that could not be validated (see Section Validation of EEG-EMG Coherence below). The center frequency (*f*_*c*_) was calculated as the geometric centroid of the volume of significant coherence along frequency:

$$f_{c}=\frac{\sum_{i\;=\;1}^{N}V_{i}f_{i}}{\sum_{i\;=\;1}^{N}f_{i}},$$

where *V*_*i*_ is a voxel of significant coherence at frequency, *f*_*i*_, and *N* is the total number of *V*_*i*_ within the binned ensemble average of EEG-EMG coherence.

#### Statistical analysis

For each measure of motor performance, we performed 2-way analysis of variance (ANOVA) with (i) the type of pacing (i.e., self- or external pacing) and (ii) the sides of the body (i.e., left or right) as factors. For the volume and center frequency of significant coherence, we performed 3-way ANOVA on the coherence between the EEG signal from C_z_ and EMG signals of the tibialis anterior and medial gastrocnemius muscles. For the 3-way ANOVA, the factors were (i) the type of pacing, (ii) muscle (i.e., tibialis anterior or medial gastrocnemius muscles), and (iii) the side of the body. To compare the volume of significant coherence among all EEG electrode locations, we performed 4-way ANOVA with (i) EEG electrode location, (ii) the type of pacing, (iii) muscle, and (iv) the side of the body as factors. If any factor showed a significant main effect in the aforementioned ANOVA, we performed *post hoc* analysis with Tukey's honestly significant difference procedure. The significant level was set to 5% for all tests.

#### Validation of EEG-EMG coherence

We used surrogate coherence to validate the experimental coherence at C_z_. For each participant, an ensemble average of coherence was calculated with shuffled pairing between EEG and EMG signals: the *i*th cycle of an EEG signal was paired with the *j*th cycle of an EMG signal, such that *i* ≠ *j* and none of the original pairing was preserved. To match the durations of paired segments of EEG and EMG signals, all segments were re-sampled to the average cycle duration. The re-sampling was performed with margins of fifty data points on either side of each segment. For each participant, 100 such ensemble averages were calculated with differently permutated pairing of EEG and EMG signals, and the average magnitude of the 100 ensemble averages was used as the surrogate coherence. To validate the experimental coherence, we examined how the shuffled pairing of signals affected the volume of significant coherence at C_z_. For each pair of experimental and surrogate coherence, their significance was determined by the same threshold value. The effects of shuffled pairing were examined using 4-way ANOVA with (i) the type of pacing, (ii) muscle, (iii) the side of the body, and (iv) shuffling (i.e., pre- or post-shuffling) as factors. From preliminary analysis, we observed that shuffling the pairing between EEG and EMG signals resulted in residual, relatively high coherence at lower frequencies (generally up to 6 Hz). Therefore, the above ANOVA was performed separately above and below 6 Hz.

#### Group average of EEG-EMG coherence

At each EEG electrode location, the magnitude of cyclical coherence was averaged among participants to yield a group average. For the group average, the threshold of significance was calculated using the average number of movement cycles completed among participants. The surrogate coherence was also averaged among participants to yield a group average.

## Results

### Kinematic data

**Figure 3:**
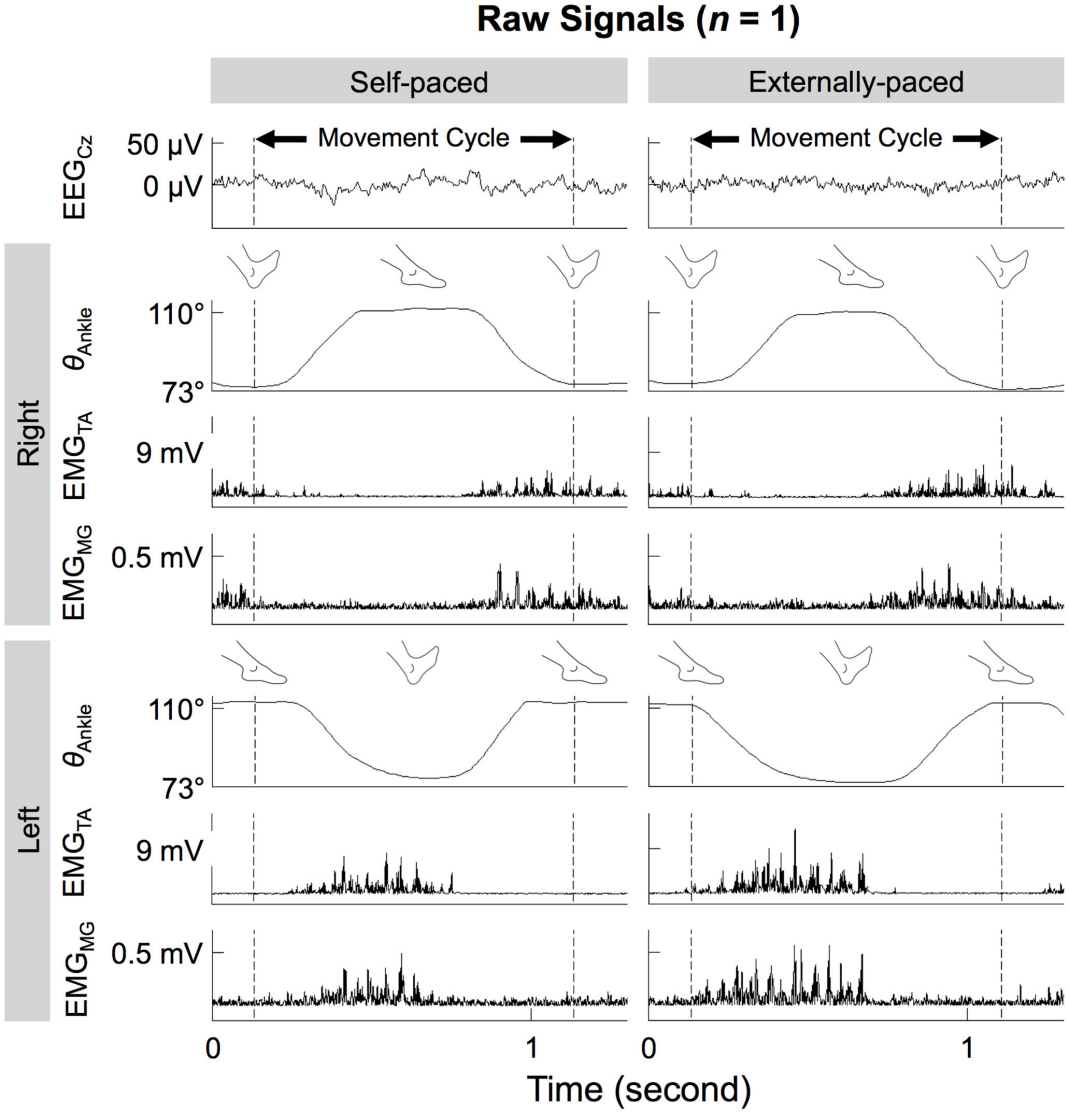
**Ankle angles (θ_**Ankle**_), EMG signals from the tibialis anterior and medial gastrocnemius muscles (EMG_**TA**_ and EMG_**MG**_), and noise-reduced EEG signal from C_**z**_ (EEG_**Cz**_) during self- and externally-paced movements**. All signals are from the same representative participant.

Figure 3 shows the time course of ankle angles within a movement cycle. During each 1-min run, the participants completed 56.6 ± 3.0 cycles. After each run, the participants rested 94.8 ± 58.8 s. The cycle duration was 1.11 ± 0.03 s. The range of motion at the ankle was 38.0 ± 6.9°, with maximum and minimum angles of 122 ± 7° and 83.8 ± 8.0°, respectively.

Neither the type of pacing nor the side of the body significantly affected the mean and standard deviation of the cycle duration and range of motion (Table 1). The effect of the type of pacing was relatively large on the standard deviation of the movement cycle duration, but the effect did not reach significance [*F*_(1, 54)_ = 3.66, *p* = 0.0611]. In other words, motor performance did not differ significantly between self- and external pacing and between left and right feet. Also, there were no significant interactions between the type of pacing and side of the body for the parameters of motor performance (Table 1).

**Table 1:** **Results of 2-way ANOVA on the intra-individual mean (μ) and standard deviation (σ) of cycle duration and range of motion**.

**Dependent Variable**		**Main Effects**		**Interaction**
		**Side of body**	**Type of pacing**	**Side of body × Type of pacing**
Cycle Duration	μ	*F*_(1, 54)_ < 0.01, *p* = 0.981	*F*_(1, 54)_ = 1.47, *p* = 0.231	*F*_(1, 54)_ = 0.00140, *p* = 0.970
	σ	*F*_(1, 54)_ = 1.49, *p* = 0.228	*F*_(1, 54)_ = 3.66, *p* = 0.0611	*F*_(1, 54)_ = 0.113, *p* = 0.738
Range of Motion	μ	*F*_(1, 54)_ < 0.01, *p* = 0.981	*F*_(1, 54)_ = 0.0119, *p* = 0.914	*F*_(1, 54)_ = 0.00714, *p* = 0.933
	σ	*F*_(1, 54)_ = 0.0401, *p* = 0.842	*F*_(1, 54)_ = 0.424, *p* = 0.518	*F*_(1, 54)_ = 0.206, *p* = 0.652

Regardless of the type of pacing, the motion-capture markers on the head were within a volume of approximately 1 cm^3^ during each movement cycle. The average cyclic linear head movements were no more than 7, 6, and 4 mm, in the anteroposterior, mediolateral, and longitudinal directions, respectively.

### EEG-EMG coherence during cyclical ankle movements

Figure 3 shows the time courses of the EEG signals from C_z_ and EMG signals from the tibialis anterior and medial gastrocnemius muscles of a representative participant. On both sides of the body, the two muscles co-contracted during dorsiflexion of the ipsilateral foot. This pattern was observed for both types of pacing.

**Figure 4:**
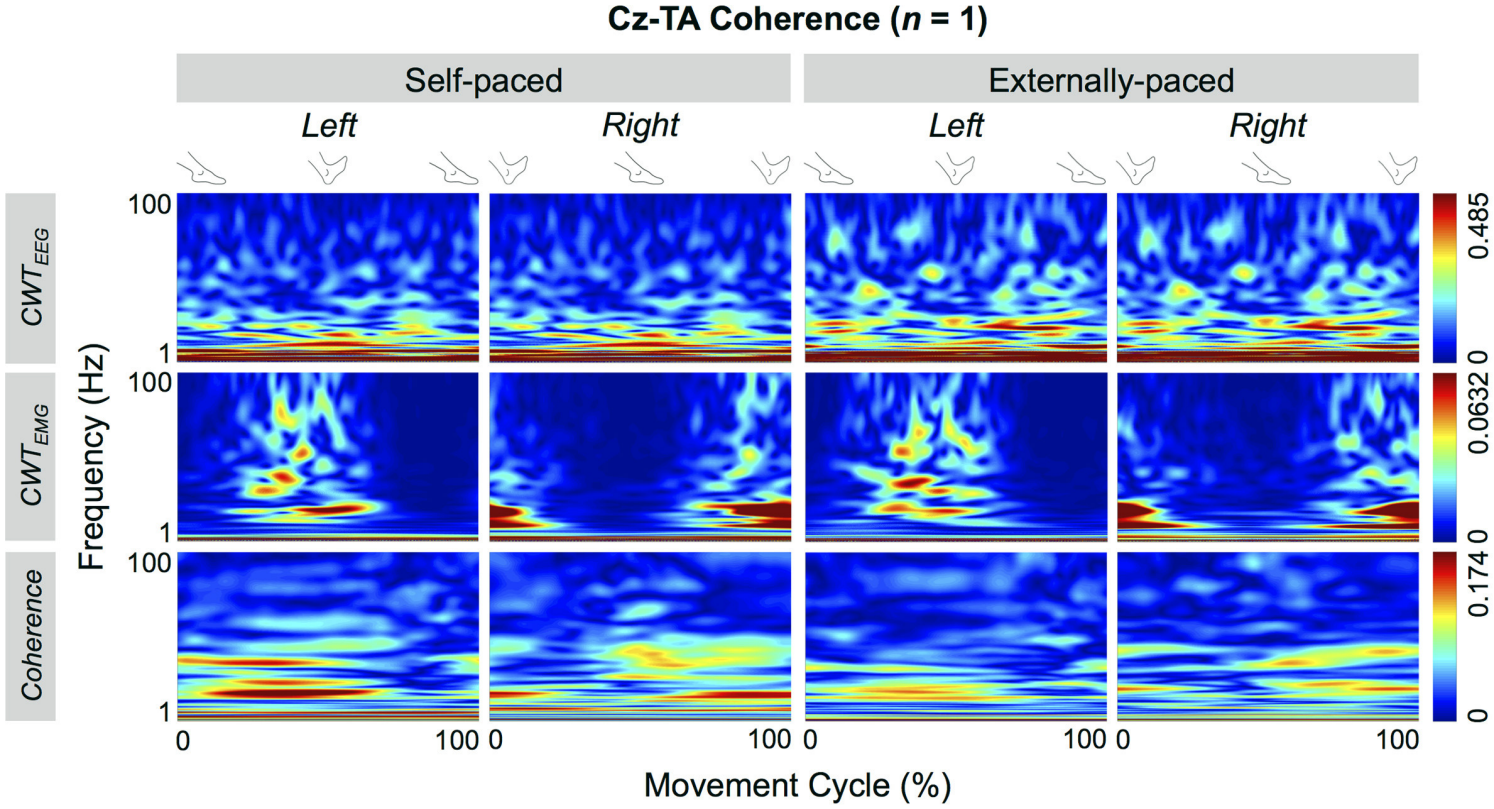
**Wavelet coherence between EEG signal from C_**z**_ and EMG signal from the tibialis anterior (TA) muscle of a representative participant**. The top two rows show continuous wavelet transforms (CWT) of the EEG and EMG signals, and the bottom row shows their coherence.

Figure 4 shows the cyclical frequency-time distributions of the EEG signal from C_z_, EMG signals from the tibialis anterior muscle, and their wavelet coherence for a representative participant. The coherence increased cyclically below 50 Hz and approximately during dorsiflexion (cf. Figure 3).

**Figure 5:**
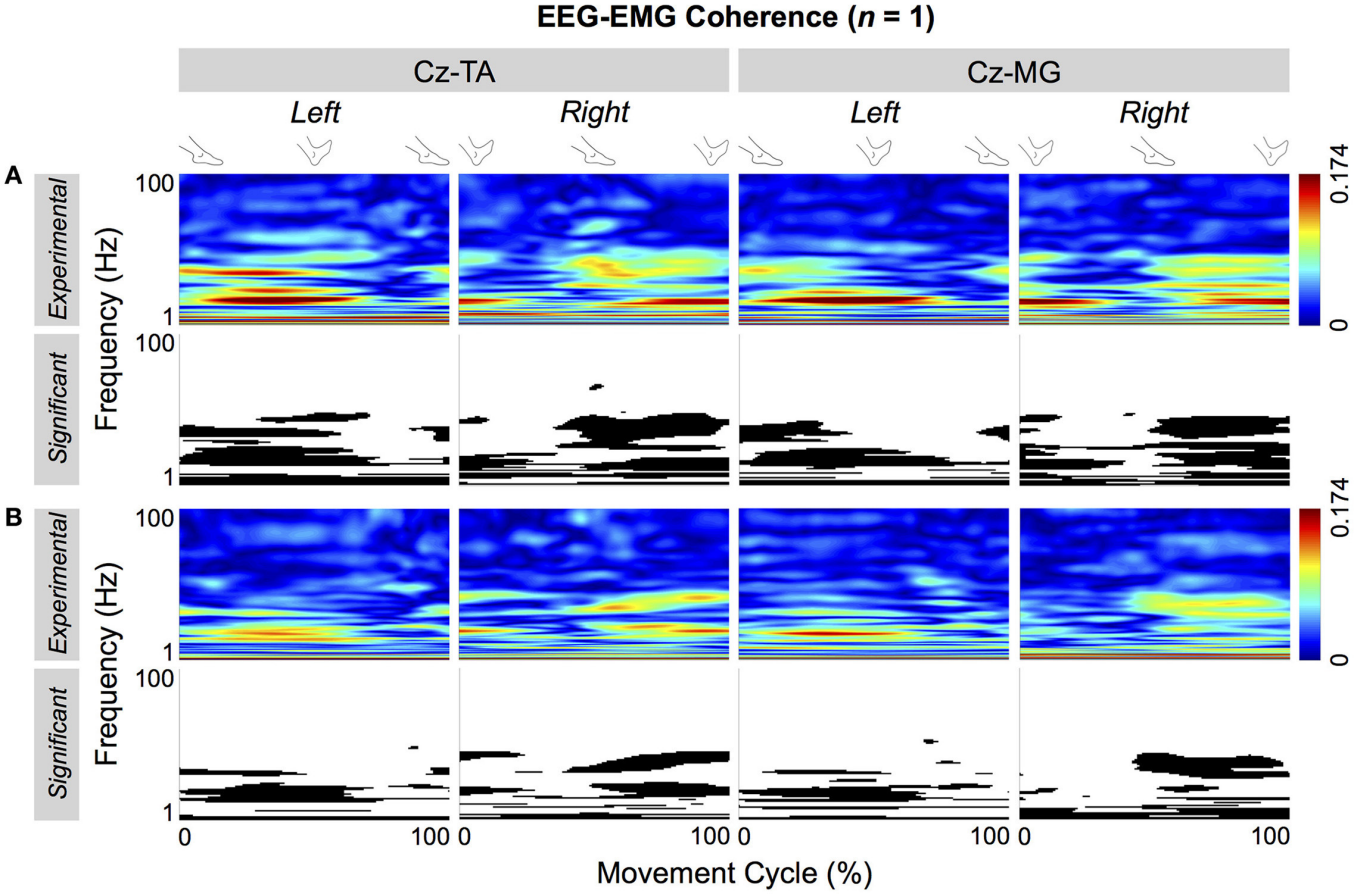
**Cyclical EEG-EMG coherence of a representative participant**. Coherence is calculated between C_z_ and the tibialis anterior (TA) and medial gastrocnemius (MG) muscles. **(A,B)** Respectively show coherence for self- and externally-paced movements. For each type of pacing, the black and white patterns in the bottom row indicate the significant portions of the patterns in the top row.

Figure 5 shows the significant portions of the cyclical wavelet coherence between C_z_ and the two muscles of a representative participant. For both types of pacing and muscles, the cyclical increase in coherence was significant. For this participant, the threshold values for significant coherence were 0.0697 and 0.0705 for self-paced and externally-paced movements, respectively, with 170 and 168 movement cycles. For the group, the thresholds of significance were 0.0705 ± 0.0031 and 0.0697 ± 0.0022 for self-paced and externally-paced movements, respectively, with 170 ± 8 and 171 ± 6 movement cycles.

**Figure 6:**
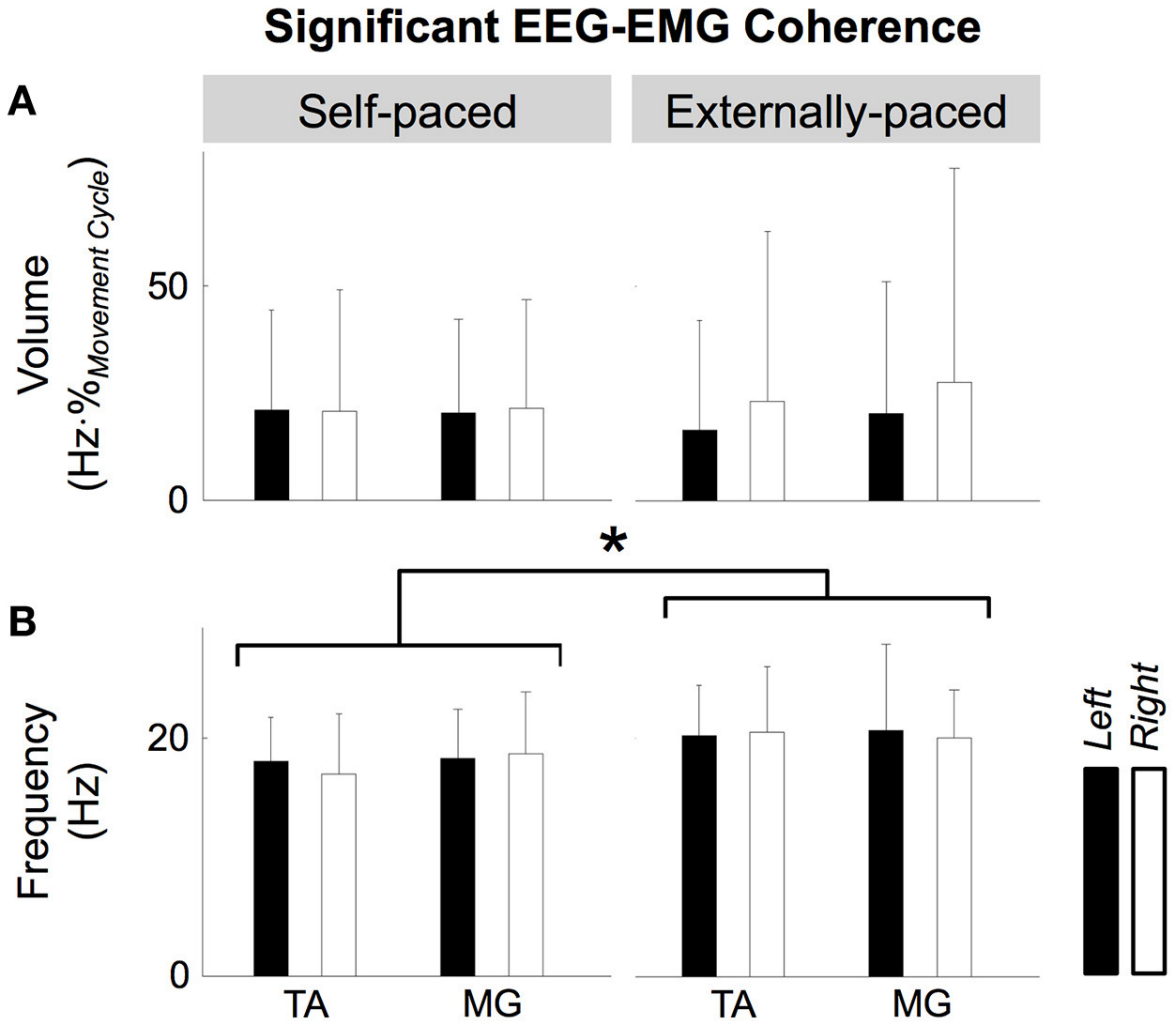
**Volume (A)** and center frequency **(B)** of significant coherence between EEG signal from C_z_ and EMG signals from the tibialis anterior (TA) and medial gastrocnemius (MG) muscles. The asterisk indicates a significant difference with a significant level of 5%.

Figure 6 shows the volume and center frequency of significant EEG-EMG coherence between C_z_ and the two muscles. The volume of coherence was not significantly affected by the type of pacing [*F*_(1, 109)_ = 0.0299, *p* = 0.863], muscle [*F*_(1, 109)_ = 0.123, *p* = 0.726], or the side of the body [*F*_(1, 109)_ = 0.398, *p* = 0.529]. The center frequency was significantly affected by the type of pacing [*F*_(1, 109)_ = 6.48, *p* = 0.0123], but not by the muscle [*F*_(1, 109)_ = 0.251, *p* = 0.618] or the side of the body [*F*_(1, 109)_ = 0.0689, *p* = 0.793]. A *post hoc* test revealed that the center frequency was higher with external pacing. None of the factors of 3-way ANOVA (i.e., type of pacing, muscle, and side of the body) interacted significantly for the volume and center frequency of significant EEG-EMG coherence (Table 2).

**Table 2:** **Interactions between the factors of 3-way ANOVA on the volume and center frequency of significant EEG-EMG coherence**.

**Dependent variable**	**Interaction**		
	**Type of pacing × Muscle**	**Type of pacing × Side of body**	**Muscle × Side of body**
Volume	*F*_(1, 09)_ = 0.125, *p* = 0.724	*F*_(1, 09)_ = 0.310, *p* = 0.579	*F*_(1, 09)_ = 0.00707, *p* = 0.933
Center Frequency	*F*_(1, 09)_ = 0.288, *p* = 0.593	*F*_(1, 09)_ = 0.00693, *p* = 0.934	*F*_(1, 09)_ = 0.0220, *p* = 0.882

**Figure 7:**
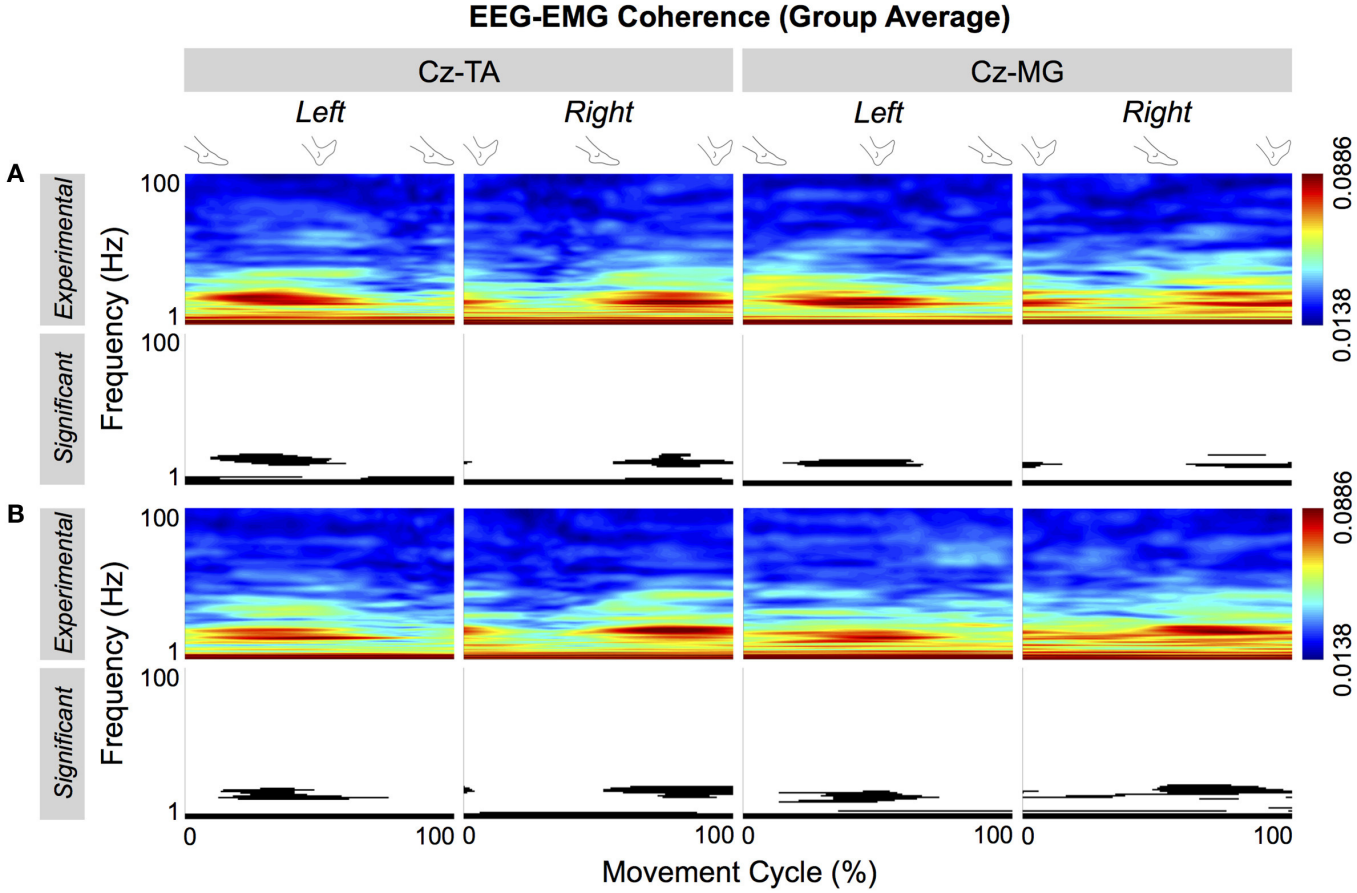
**Group average of cyclical EEG-EMG coherence**. Coherence is calculated between C_*z*_ and the tibialis anterior (TA) and medial gastrocnemius (MG) muscles. **(A,B)** Respectively show coherence for self- and externally-paced movements. For each type of pacing, the black and white patterns in the bottom row indicate the significant portions of the patterns in the top row.

Figure 7 shows the group average of the cyclical EEG-EMG coherence. The thresholds of significance for the group average were 0.0694 and 0.0693 for self-paced and externally-paced movements, respectively. In the group average, only the coherence near the β band became cyclically significant, indicating that these patterns were most common among the participants regardless of the muscle or type of pacing.

**Figure 8:**
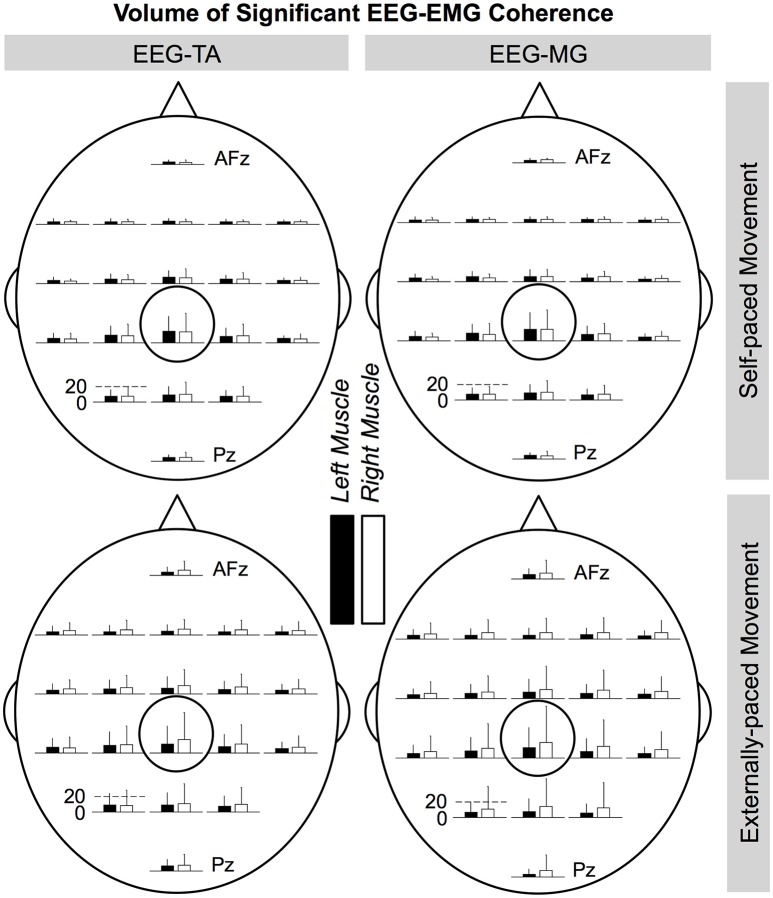
**Cortical distributions of significant coherence between EEG signals and EMG signals from the tibialis anterior (TA) and medial gastrocnemius (MG) muscles (group data)**. C_z_ is circled. At each electrode location, the bar indicates the volume of significant coherence, measured in Hz multiplied by the percentage of movement cycle (Hz·%_*Movement Cycle*_). The scale of the vertical axis is the same for all distributions. Error bars indicate inter-individual standard deviations.

**Figure 9:**
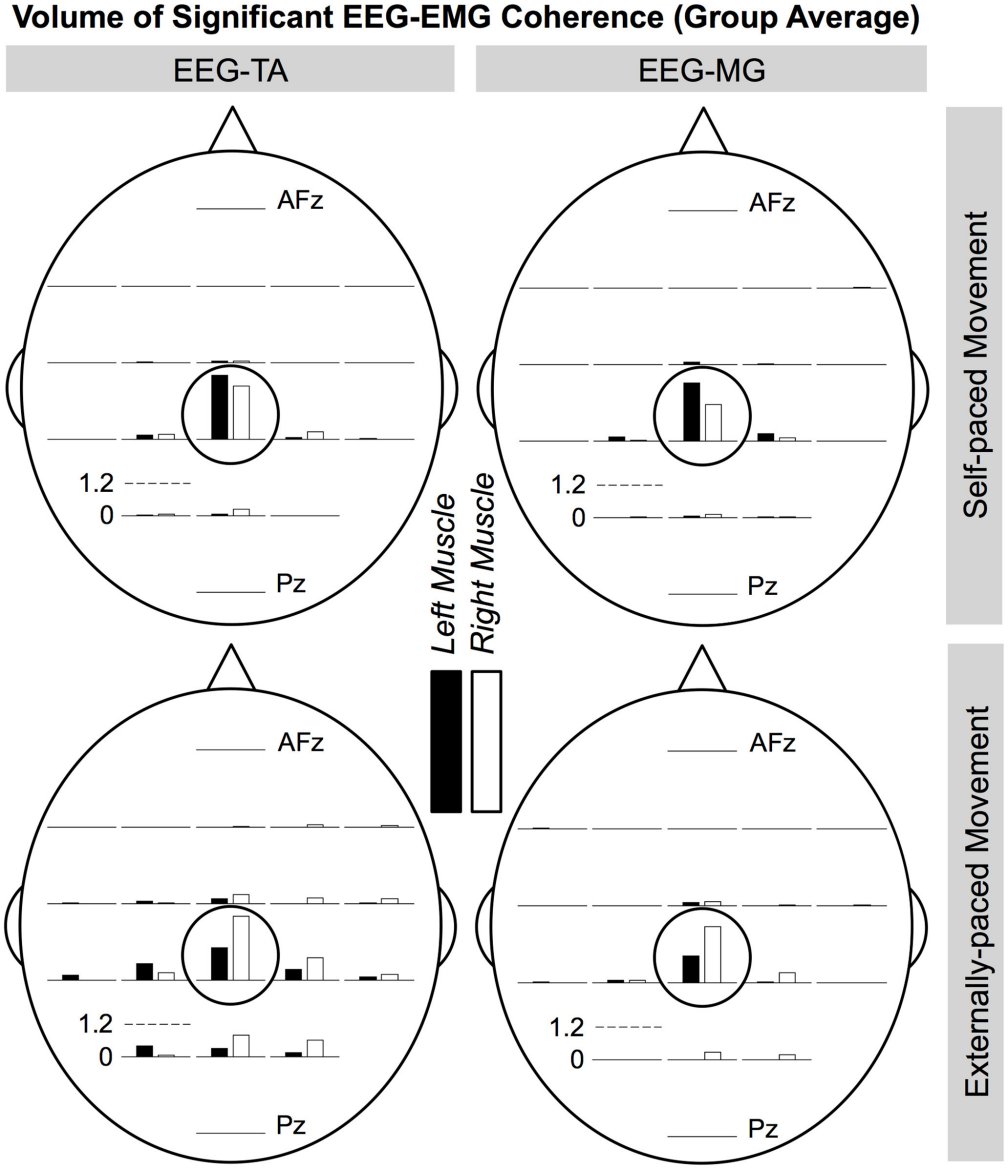
**Cortical distributions of significant EEG-EMG coherence (group average) between EEG signals and EMG signals from the tibialis anterior (TA) and medial gastrocnemius (MG) muscles**. C_z_ is circled. At each electrode location, the bar indicates the volume of significant coherence, measured in Hz multiplied by the percentage of movement cycle (Hz·%_*Movement Cycle*_). The scale of the vertical axis is the same for all distributions.

Figures 8, 9 show the cortical distributions of the volume of significant coherence for group data and group average, respectively. The average volume of significant coherence was largest at C_z_ regardless of the muscle or the type of pacing. Based on 4-way ANOVA, the volume of significant EEG-EMG coherence was significantly affected by the EEG electrode location [*F*_(19, 2237)_ = 5.36, *p* < 0.001]. A *post hoc* test showed that the volume at C_z_ was significantly larger than those at all other electrode locations, except for C_1_, C_2_, and CP_z_. The volumes did not differ significantly among other electrode locations. The volume was also significantly affected by the type of pacing [*F*_(1, 2237)_ = 11.9, *p* < 0.001] and side of the body [*F*_(1, 2237)_ = 5.90, *p* = 0.0152]. *Post hoc* tests showed that the volumes were significantly larger with external pacing and for the right side. The volume was not significantly affected by the muscle [*F*_(1, 2237)_ = 2.24, *p* = 0.135].

### Validation of EEG-EMG coherence

**Figure 10:**
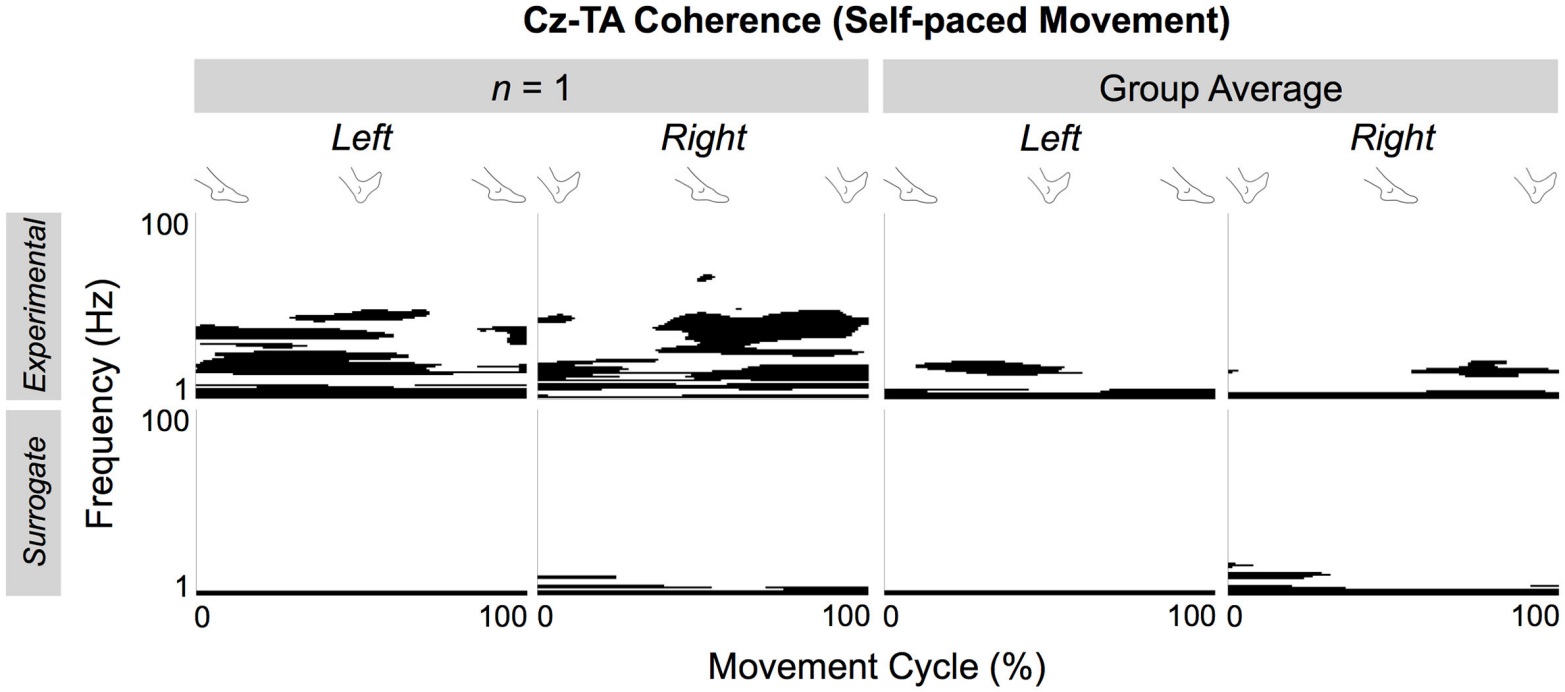
**Significant portions of experimental and surrogate EEG-EMG coherence of a representative participant and group average**. Coherence is calculated between C_z_ and the tibialis anterior (TA) muscles during self-paced ankle movements.

**Figure 11:**
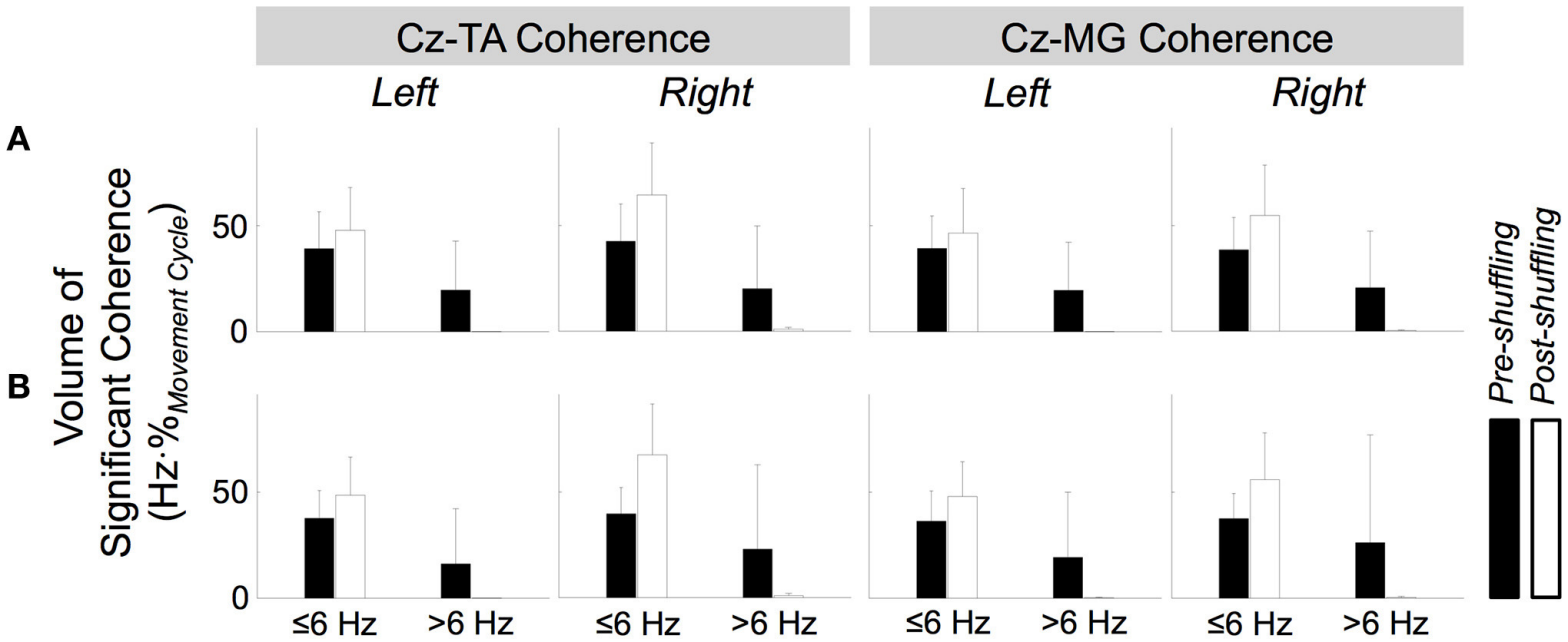
**Effects of shuffled pairing between EEG signal at C_**z**_ and EMG signals from the tibialis anterior (TA) and medial gastrocnemius (MG) muscles. (A,B)** Respectively show volumes of significant coherence for self- and externally-paced movements. The error bars indicate inter-individual standard deviations.

Figure 10 shows the significant portions of the experimental and surrogate EEG-EMG coherence (top and bottom rows, respectively) for a representative participant (left two columns) and group average (right two columns). For the representative participant, the surrogate coherence was only significant at lower frequencies, and shuffled pairing of EEG and EMG signals abolished the cyclical patterns of significant coherence that were observed in the experimental coherence. This phenomenon was also observed in the group average. The low-frequency coherence and the absence of cyclical coherence at higher frequencies were observed in the surrogate coherence for both muscles and types of pacing (Figure 11).

Figure 11 shows how the volume of significant coherence changes above and below 6 Hz due to shuffled pairing of EEG at C_z_ and EMG signals. Above 6 Hz, the volume of significant coherence was significantly affected by shuffling [*F*_(1, 221)_ = 45.3, *p* < 0.001] but not by the muscle [*F*_(1, 221)_ = 0.0539, *p* = 0.817], side of the body [*F*_(1, 221)_ = 0.531, *p* = 0.467], or type of pacing [*F*_(1, 221)_ = 0.0458, *p* = 0.831]. A *post hoc* test revealed that the volume above 6 Hz became smaller (and almost negligible) after shuffling. These results validate that, above 6 Hz, the cyclical increase in experimental coherence was not due to the cyclical increase in either EEG or EMG signal alone.

Below 6 Hz, the volume was significantly affected by shuffling and the side of the body [*F*_(1, 221)_ = 41.3, *p* < 0.001 and *F*_(1, 221)_ = 9.06, *p* = 0.00292, respectively] but not by the muscle [*F*_(1, 221)_ = 2.56, *p* = 0.111] or type of pacing [*F*_(1, 221)_ = 0.0100, *p* = 0.920]. *Post hoc* tests revealed that the volume was larger after shuffling and for the right limb.

Above 6 Hz, none of the factors of 4-way ANOVA interacted significantly. Below 6 Hz, only shuffling and the side of the body interacted significantly [*F*_(1, 221)_ = 5.82, *p* = 0.0166], probably indicating that the post-shuffle increase was greater on the right side.

## Discussion

### EEG-EMG coherence during bilateral, cyclical ankle movements

During the ankle movements, we observed a cyclical increase in the EEG-EMG coherence that approximately coincided with the co-contraction of the tibialis anterior and medial gastrocnemius muscles (Figures 5, 7). We also found that the EEG-EMG coherence occurred near the β band and was largest over C_z_ regardless of the muscle, side of the body, or type of pacing (Figures 8, 9). Furthermore, the cyclical increase in coherence was validated using surrogate coherence (Figures 10, 11).

Most previous studies have reported corticomuscular coherence during sustained, weak muscle contractions (Brown et al., 1998; Kristeva-Feige et al., 2002; Omlor et al., 2007, 2011; Masakado and Nielsen, 2008; Chakarov et al., 2009; Johnson et al., 2011; Ushiyama et al., 2011b; McClelland et al., 2012; Ushiyama, 2013; Trenado et al., 2014). Furthermore, a few studies have shown that, corticomuscular coherence occurs consistently throughout sustained, weak isometric or isotonic contractions (Kilner et al., 1999; Masakado and Nielsen, 2008). These findings suggest that corticomuscular coherence occurs during periods of increased muscle activation. Indeed, we observed a cyclical increase in EEG-EMG coherence that approximately coincided with the co-contraction of two leg muscles. Such a pattern is similar to the cyclical increase in coherence that occurs during treadmill walking (Petersen et al., 2012) as well as the cyclical increase in the activity of the sensorimotor cortex during robot-assisted walking (Wagner et al., 2012, 2014; Seeber et al., 2014, 2015; Storzer et al., 2016), pedaling on a stationary bike (Storzer et al., 2016), and rhythmic finger movements (Seeber et al., 2016).

Some studies have shown that corticomuscular coherence disappears between two periods of sustained contractions (i.e., while the level of contraction is increased from one period to the next) (Kilner et al., 2000; Riddle and Baker, 2006; Masakado and Nielsen, 2008). Such findings may suggest that corticomuscular coherence does not occur during movements. However, multiple studies have observed corticomuscular coherence during various movements: treadmill walking (Petersen et al., 2012), slowly increasing dorsiflexion of the foot (Masakado and Nielsen, 2008), slow self-paced wrist extension and flexion around 0.2 Hz (Brown et al., 1998), and index finger flexion against dynamic forces (Chakarov et al., 2009; Trenado et al., 2014). Thus, the absence of coherence between periods of sustained contractions may be task-specific.

### Possible mechanism of EEG-EMG coherence

Coherence quantifies whether two signals can be the input and output of a linear system. In this study, we assumed that an input-output relationship existed between surface EEG and EMG signals. We further assumed that the EEG signal from C_z_ primarily reflected the postsynaptic potentials on the apical dendritic tufts of the pyramidal neurons in the primary motor cortex (Olejniczak, 2006; Kirschstein and Köhling, 2009; Bucci and Galderisi, 2011; Buzsáki et al., 2012) and that these pyramidal neurons received predominantly excitatory input (Spruston, 2008). Lastly, because pyramidal neurons that connect monosynaptically to the α motor neurons are concentrated in the primary motor cortex (Maertens De Noordhout et al., 1999; Kalaska and Rizzolatti, 2013), the most appropriate scenario for EEG-EMG coherence may be monosynaptic corticomotoneuronal recruitment via the corticospinal tracts. If more complex circuits are involved, it becomes less likely that the system between the primary motor cortex and the activated muscle is linear.

The cyclical increase in C_z_-EMG coherence near the β band suggests that the motor units had been recruited at these frequencies. The motor unit recruitment in the β band has been suggested by the intramuscular coherence in the tibialis anterior muscle that occurs during the swing phase of treadmill walking (Halliday et al., 2003; Hansen et al., 2005). Furthermore, the absence of such intramuscular coherence in individuals with incomplete spinal cord injury implies the supraspinal origin of the recruitment (Hansen et al., 2005). Lastly, it has been demonstrated experimentally (Negro and Farina, 2011) and computationally (Stegeman et al., 2010; Negro and Farina, 2011; Farina et al., 2014) that the frequency of recruitment can be linearly transmitted from presynaptic input to the motoneuronal group that receives the input. There has been some criticism against overestimating the percentage of motor units that are synchronized by common input. With a more statistically rigorous method, De Luca and Kline (2014) found that only 50% of the motor units are synchronized by common input. However, the tibialis anterior and medial gastrocnemius muscles are innervated by over 400 and 500 α motor neurons, respectively (Enoka and Pearson, 2013), and less than 10 motor units are necessary to show clear corticomuscular coherence (Negro and Farina, 2011). Thus, it is likely that enough motor units will be synchronized by common input to show corticomuscular coherence during weak muscle contractions.

Although corticomuscular coherence suggests corticospinal muscle activation, it does not specify the source of the synchronous input to the primary motor cortex. Witham et al. (2011) have suggested that, during a precision grip task, afferent feedback may be involved in corticomuscular coherence. However, the origin of the synchronous input could not be determined definitively for this study.

Regardless of where the synchronous input originates, the observed EEG-EMG coherence suggests that the primary motor cortex contributes to the control of simple cyclical ankle movements. In cats, corticospinal contribution appears to modify the basic patterns of locomotion for skillful movements (e.g., obstacle avoidance; Drew et al., 2004). However, the skillful gait modifications are thought to occur through the integration of cortical signals into the pattern-generating (probably spinal) circuit (Drew et al., 2004). Because such processing may be complex (and possibly less linear), the corticospinal contribution that is reflected in EEG-EMG coherence is probably less relevant to the ongoing skillful modification of cyclical movements but more relevant to specific requirements of the movement: maintaining a constant frequency and bilaterally coordinating the feet. The role of the human primary motor cortex may be similar in bipedal locomotion, during which the above requirements also apply.

### EEG-EMG coherence in medial gastrocnemius muscles

We hypothesized that EEG-EMG coherence would be observed for the tibialis anterior muscles but not for the medial gastrocnemius muscles. This hypothesis was unsubstantiated: C_z_-EMG coherence was similarly observed in both muscles (Figures 5, 7) during their co-contraction (Figure 3). This finding suggests that the primary motor cortex participates in the control of both agonist and antagonist muscles during cyclical ankle movements.

In the adopted posture (Figure 1), we expected the ankle movements to require predominantly the tibialis anterior muscles, as dorsiflexion had to be performed against gravity. Conversely, we did not expect the movements to require much contraction of the medial gastrocnemius muscles, as plantarflexion was aided by gravity and could be achieved partially through relaxing the dorsiflexors. Indeed, the amplitude of EMG signals was much smaller for the medial gastrocnemius muscles than for the tibialis anterior muscles (Figure 3). However, we did not expect the medial gastrocnemius muscles to weakly co-contract with the tibialis anterior muscles during dorsiflexion and show coherence with the primary motor cortex.

Corticomuscular coherence has been observed for co-contracting agonist and antagonist muscles during sustained isometric elbow flexion (Bayram et al., 2015). During elbow flexion, the antagonist shows lower magnitude of corticomuscular coherence compared to the agonists (Bayram et al., 2015). In this study, we found that the co-contracting agonist and antagonist (i.e., the tibialis anterior and medial gastrocnemius muscles, respectively) showed EEG-EMG coherence of comparable magnitude. The co-contraction of the medial gastrocnemius muscle may contribute to the postural control of the foot. If so, our findings suggest that the primary motor cortex dynamically participates in the postural control of the foot as well as locomotive actions.

### Effect of aural pacing on EEG-EMG coherence

Previous studies suggest that corticomuscular coherence is affected by the attention or effort in performing a precise motor task. For example, coherence is greater during isotonic contraction than isometric contraction (Masakado and Nielsen, 2008), with better performance to match a target force during isometric contraction (Kristeva et al., 2007), when greater effort is required to transition into isometric contraction (Omlor et al., 2011), during isometric contraction of a fatigued muscle (Ushiyama et al., 2011a), when a dynamic force has to be counteracted by a finger to maintain its position static (Chakarov et al., 2009; Trenado et al., 2014), when a greater digit displacement is required during a precision grip task (Kilner et al., 2000; Riddle and Baker, 2006), and when isometric contraction is mechanically perturbed (McClelland et al., 2012). Conversely, corticomuscular coherence decreases during isometric contraction when the effort or attention is reduced by a concurrent cognitive task (Kristeva-Feige et al., 2002; Johnson et al., 2011) or when the required precision of contraction is reduced (Kristeva-Feige et al., 2002). Thus, corticomuscular coherence may be linked to the degree of effort or attention in achieving specified performance.

Based on the assumption that rhythmic aural pacing would increase the participants' attention to the movement, we hypothesized that external pacing would increase the magnitude of EEG-EMG coherence. Additional evidence also supported this hypothesis, as rhythmic aural pacing can (i) make individual movement cycles more consistent through auditory entrainment (Thaut et al., 1998a,b) and (ii) increase the contributions of cortical activities to motor control by evoking periodic fields in the primary auditory cortex (Fujioka et al., 2012). However, our findings did not support the above hypothesis, as the type of pacing did not significantly affect the magnitude of coherence at C_z_ (Figure 6). Therefore, in the case of simple cyclical movements, rhythmic aural pacing may not significantly improve attention to the task and increase the degree of corticospinal muscle activation. However, the lack of task-dependence may be attributed to the particular sequence of external and self-pacing that we used (i.e., externally- and self-paced movements alternated with external pacing always being performed first). This sequence may have affected the self-paced movements, as participants could remember the rhythm of the aural pacing from the previous run. The magnitude of coherence may have differed had the participants first performed the ankle movements at a self-selected pace and external pacing was applied at the self-selected pace.

Although the magnitude of coherence was unaffected, its frequency was slightly but significantly increased by external pacing for both muscles (Figure 6). Omlor et al. (2007) have reported an increase in the frequency of peak coherence due to multisensory integration. In their study, participants were asked to maintain the position of a manipulandum static against sinusoidal mechanical perturbation while visually monitoring the performance (Omlor et al., 2007). For this task, the frequency of peak coherence was higher than the frequency for isometric contractions: a shift from 15–30 to 30–45 Hz (Omlor et al., 2007). In this study, the shift in frequency was smaller than what Omlor et al. reported, but the degree of sensorimotor integration was also arguably less. Therefore, the observed increase in frequency with external pacing may have some physiological relevance if we assume that the magnitude of shift in frequency is proportional to the degree of sensorimotor integration.

## Conclusion

We have shown that cyclical increase in corticomuscular coherence, which has been observed during walking, also occurs during simple bilateral, cyclical ankle movements. One possible mechanism for such coherence is corticomuscular communication, in which the primary motor cortex participates in the control of movement. However, additional studies are needed to identify what cortical and subcortical interactions cause corticomuscular coherence. Additional studies are also needed to delineate the functional role of the primary motor cortex during bilateral cyclical movements such as walking. However, for the ankle movements, with fewer functional requirements than walking, the observed coherence suggests that the primary motor cortex may participate in (i) maintaining a constant movement frequency, (ii) bilaterally coordinating the feet, or (iii) stabilizing the posture of the foot through weak co-contraction of the antagonist muscle.

## Ethics statement

This study was carried out in accordance with the recommendations of the University Health Network Research Ethics Board, Toronto, Canada, with written informed consent from all subjects. All subjects gave written informed consent in accordance with the Declaration of Helsinki. The protocol was approved by the University Health Network Research Ethics Board.

## Author contributions

TY, KM, and KZ designed the experiment. KZ also provided technical consulting. TY performed the experiments and analyzed the data. TY, KM, RC, and MP interpreted the data. TY drafted the manuscript. KZ, RC, and MP edited the manuscript. TY and KM revised the manuscript.

## Funding

TY was supported by the Toronto Rehabilitation Institute Student Scholarship from the University of Toronto and the CREATE Academic Rehabilitation Engineering Fellowship from the Natural Sciences and Engineering Research Council of Canada. This work was partially supported by a Canadian Institutes of Health Research grant (OMH131582; KM). The authors also acknowledge the support from the Toronto Rehabilitation Institute - University Health Network, Dean Connor and Maris Uffelmann Donation, and Natural Sciences and Engineering Research Council Discovery Grant (#249669; MP).

### Conflict of interest statement

The authors declare that the research was conducted in the absence of any commercial or financial relationships that could be construed as a potential conflict of interest.
